# T6SS Mediated Stress Responses for Bacterial Environmental Survival and Host Adaptation

**DOI:** 10.3390/ijms22020478

**Published:** 2021-01-06

**Authors:** Kai-Wei Yu, Peng Xue, Yang Fu, Liang Yang

**Affiliations:** School of Medicine, Southern University of Science and Technology, Shenzhen 518055, China; yukw2020@mail.sustech.edu.cn (K.-W.Y.); pengxueubc@163.com (P.X.); fuy@sustech.edu.cn (Y.F.)

**Keywords:** bacteria, stress response, signaling interference, T6SS

## Abstract

The bacterial type VI secretion system (T6SS) is a protein secretion apparatus widely distributed in Gram-negative bacterial species. Many bacterial pathogens employ T6SS to compete with the host and to coordinate the invasion process. The T6SS apparatus consists of a membrane complex and an inner tail tube-like structure that is surrounded by a contractile sheath and capped with a spike complex. A series of antibacterial or antieukaryotic effectors is delivered by the puncturing device consisting of a Hcp tube decorated by the VgrG/PAAR complex into the target following the contraction of the TssB/C sheath, which often leads to damage and death of the competitor and/or host cells. As a tool for protein secretion and interspecies interactions, T6SS can be triggered by many different mechanisms to respond to various physiological conditions. This review summarizes our current knowledge of T6SS in coordinating bacterial stress responses against the unfavorable environmental and host conditions.

## 1. Introduction

**Figure 1:**
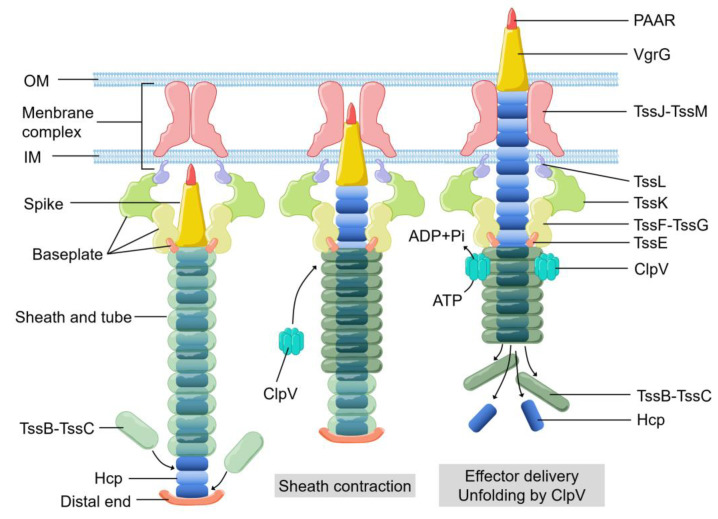
The basic components, structural assembly, and effector delivery of the T6SS apparatus. OM, outer membrane. IM, inner membrane.

Bacteria can use secretion systems to transport individual proteins, as well as DNA–protein and protein–protein complexes into adjacent cells or the external medium. These secretion systems are critical for bacterial survival and adaptation to complex environmental conditions via their diversified secreted effectors, which are required for nutrition uptake, toxin delivery, cell-to-cell communication, and interspecies competition. Nine secretion systems (T1SS, T2SS, T3SS, T4SS, T5SS, T6SS, T7SS, T8SS (curli/Csg), and T9SS) have been identified in Gram-negative and Gram-positive bacteria [1,2,3,4]. Among them, the type VI secretion system (T6SS) (Figure 1), a complex contractile nano-machine similar to bacteriophage tail in structure, retains one of the most complicated secretion mechanisms [5]. T6SS is a needle-like multiprotein machine which is a member of contractile injection systems. Its structure consists of the membrane complex, the baseplate structure, and the tail tube sheath complex [6,7,8]. ClpV, a type of hexameric AAA+ ATPase, pulls the exposed N terminus of TssC and releases it from the contracted sheaths to replenish the pool of sheath subunits for starting a new cycle of assembly [9,10,11]. VgrG trimer recruits the hemolysin coregulated protein (Hcp) to become polymerized as the inner tube [5,12]. The type Ⅵ secretion system (T6SS) of bacteria was first detected in Rhizobium leguminosarum in 2003 [13,14]. In the next seventeen years, it was found that at least 1/4 Gram-negative bacteria species contain T6SS [15,16]. T6SS is often involved in multiple processes related to bacterial virulence [17,18,19,20,21]. To cope with external pressures, bacteria use T6SS to secret toxins into the external environment [3,22]. Moreover, T6SS has been shown to attack bacterial competitors [23,24,25], or to defeat the host defense mechanisms [26,27], in order to colonize a host niche and/or survive in competition. In this review, we discuss the current findings on the T6SS-mediated stress responses to reactive oxygen species, temperature, pH during competition and invasion processes.

## 2. Metal Ion Uptake for ROS Stress Response

Both the host immunity and the environmental factors such as heavy metals and antibiotics will lead to increased reactive oxygen species (ROS) levels in bacterial pathogens significantly [2]. The formation of ROS (e.g., hydrogen peroxide, hydroxyl radical, and superoxide) is inevitable in an oxygen-rich environment [28,29]. Other factors causing the generation of ROS include peptidoglycan recognition proteins [30], antimicrobial compounds with bactericidal activity [31], and attacks from competing bacteria and bacteriophages [32]. Elevated levels of ROS in cells will cause oxidative damage to DNA, proteins, lipids, and other macromolecules [33]. Bacteria are well known to respond to oxidative stress with the help of some regulatory proteins, like SoxR, SoxS, OxyR, or ZntR, which coordinate specific ROS stress tolerance mechanisms [34,35,36]. For example, the synthesis of antioxidant enzymes (e.g., peroxidase, glutaredoxin, uperoxide dismutase, thioredoxin) and small molecular weight antioxidants (e.g., β-carotene, tripeptide glutathione, vitamin C, and vitamin E) can be induced via these regulatory proteins to combat the adverse effects of ROS [33,37,38].

**Figure 2:**
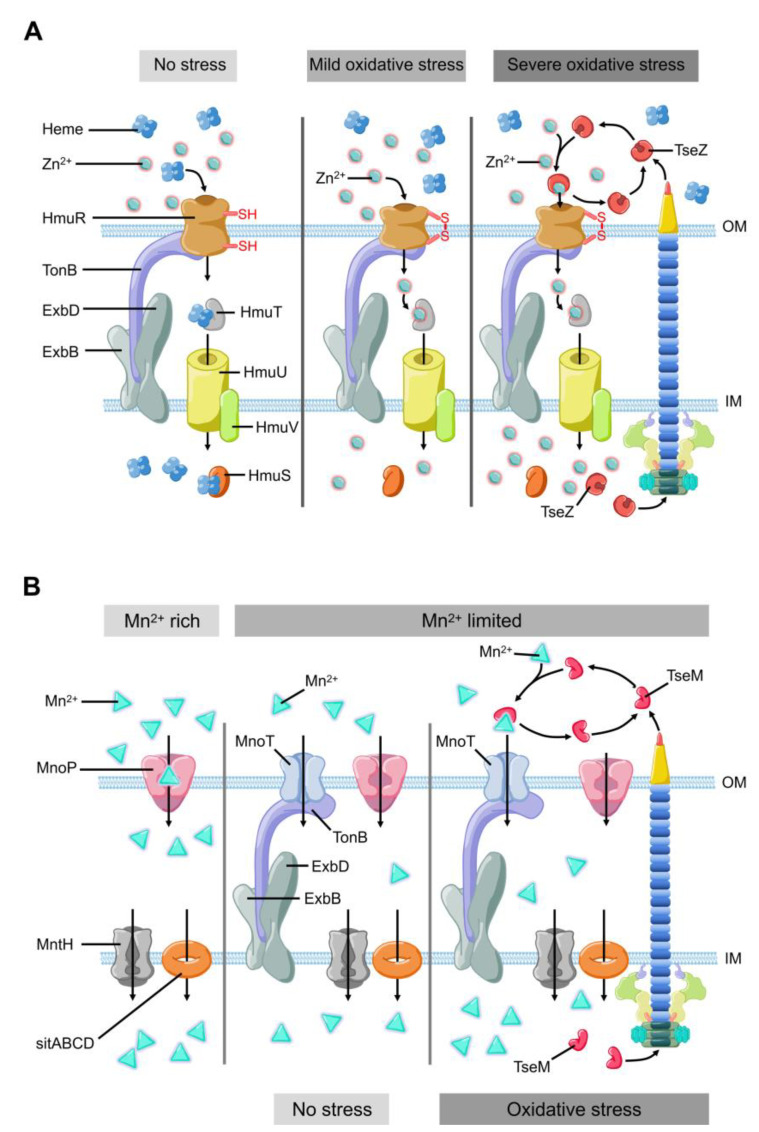
The model of T6SS-mediated stress response to environmental pressure. (**A**) *B. thailandensis* T6SS effector TseZ as a proteinaceous zincophore interacts with heme transporter HmuR to acquire zinc, which facilitates Zn^2+^ transportation to resist oxidative stress. HmuR forms intramolecular disulfide bond to transport zinc instead of heme under oxidative stress. (**B**) *B. thailandensis* T6SS effector TseM transports Mn^2+^ to resist oxidative stress with the help of outer membrane transporter MnoT. TseM is secreted into the extracellular milieu to scavenging Mn^2+^ and deliver it to MnoT when bacteria encounter oxidative stress.

In recent years, it has been revealed that T6SS is involved in the process of oxidative stress response in bacterial pathogens, such as *Vibrio anguillarum* [39], *Burkholderia thailandensis* [40], Enterohemorrhagic *E. coli* [41], and *Yersinia pseudotuberculosis* [42]. Metal ions, such as zinc (Zn^2+^) and manganese (Mn^2+^), can be used as structural components or cofactors of antioxidant enzymes [35]. These metal ions also participate in the formation of antioxidant complexes [43,44,45,46]. Both metals help bacteria maintain redox balance and eliminate ROS [38]. Zinc (Zn^2+^) is an essential nutrient that participates in several mechanisms to maintain the redox homeostasis of bacteria, for instance, as a cofactor of superoxide dismutase [45,47]. Even though there are already well-studied systems involved in metal ion uptake and transportation, Si and colleges recently found that *B. thailandensis* employs T6SS to export TseZ as a Zn^2+^-binding effector under the condition of oxidative stress [48] (Figure 2A). The wild-type *B. thalandensis* could increase its survival rate and decrease the level of ROS by importing zinc ions when it was exposed to exogenous oxidative stress. The survival rate of *B. thalandensis clpv4* mutant, which is unable to import zinc ions via HmuR, was lower than the wild-type strain and the mutant accumulated higher level of intracellular ROS than the wild-type strain [48]. This group demonstrated that T6SS effector TseZ interacts with HmuR (the outer membrane heme transporter) and further showed that the HmuRSTUV system is involved in the acquisition of Zn^2+^ under oxidative stress conditions [48]. HmuR is a type of dual-functional transporter which is regulated by redox [48]. HmuR transports heme iron under normal conditions and forms an intramolecular disulfide upon sensing oxidative stress [48]. The formation of the disulfide bond leads to a conformational change, which allows HmuR to bind TseZ, and more efficiently transfers the chelated Zn^2+^ into the cell [48,49]. This is a fine-tuned mechanism that allows *B. thailandensis* to response to different levels of oxidative stress [48]. In *Y. pseudotuberculosis*, the MarR family transcriptional regulator HpaR [50,51] directly binds the promoter of T6SS to upregulate its expression. T6SS can secrete YezP (a zinc-binding protein substrate) which binds extracellular Zn^2+^ and transports it into *Y. pseudotuberculosis* [42]. When wild-type *Y. pseudotuberculosis* was inoculated into C57BL/6 mice, the mortality rate of the mice exceeded 50% within two weeks. In contrast, the survival rate of mice infected by T6SS or *yezP*-deficient mutant strains increased significantly [42]. This means that the *Y. pseudotuberculosis* mutant lacking T6SS or *yezP* has defective virulence in the process of infection in mice. T6SS is involved in the transportation of zinc ions and is essential to increase the survival rate of bacteria during host infection [42]. Compared with the wild-type *Y. pseudotuberculosis* strains, the strains lacking *hpaR* showed lower level of virulence when it infects mice, which further confirmed that HpaR helps *Y. pseudotuberculosis* to coordinate stress response and adapt to the host environment [43]. Therefore, HpaR positively regulates T6SS to modulate the antioxidant activity of *Y. pseudotuberculosis*, and the uptake of divalent zinc ion via a yet unknown mechanism against oxidative stress [43].

Manganese (Mn^2+^) is another important micronutrient transition metal involved in many biochemical processes, especially in the process of resisting oxidative stress [44,46]. Mn^2+^ can be used as a cofactor or as a substitute of iron in certain iron-containing enzymes that reduce the ROS damage to bacteria [44,49,52,53]. In mammalian hosts, Mn^2+^ are strictly restricted by the mechanism of nutritional immunity [54,55]. Bacteria also have several strategies to acquire manganese, including ATP-binding cassette family transporter [56], Mn2+-selective channel protein [57], and natural resistance-associated macrophage protein family transporter [58,59]. Recently, Si and colleagues reported that T6SS helps cells acquire manganese under oxidative stress in *B. thailandensis* [40]. Compared with the wild-type strain, the survival rate of *B. thailandensis* T6SS *clpV4* mutant strains under oxidative stress was decreased significantly in manganese-rich environment [40]. This difference in survival rate is regulated by OxyR (a conservative regulator of oxidative stress) via an Mn^2+^- binding T6SS effector [40,42]. OxyR is known as an oxidative stress regulator that controls the expression of many important genes to resist oxidative stress, such as *ahpCF*, *ccpA*, *dps*, *goRA*, *grxA*, *katG*, and *oxyS* [60,61,62,63,64]. Under oxidative stress condition, *B. thailandensis* T6SS secretes TseM (Mn^2+^-binding effector) (Figure 2B), which binds Mn^2+^ to promote its uptake through MnoT (a TonB-dependent outer membrane transporter). Facing the oxidative stress from cumene hydroperoxide, compared with *clpV4* mutant strains, wild-type strains exhibit higher survival rates, higher concentration of Mn^2+^ and lower intracellular ROS levels [40]. Thus, T6SS-mediated manganese uptake alleviates the attack from ROS, which significantly improves the survival rate of *B. thailandensis* under oxidative stress [40].

## 3. Adaptation to Changes in Temperature and pH

**Figure 3:**
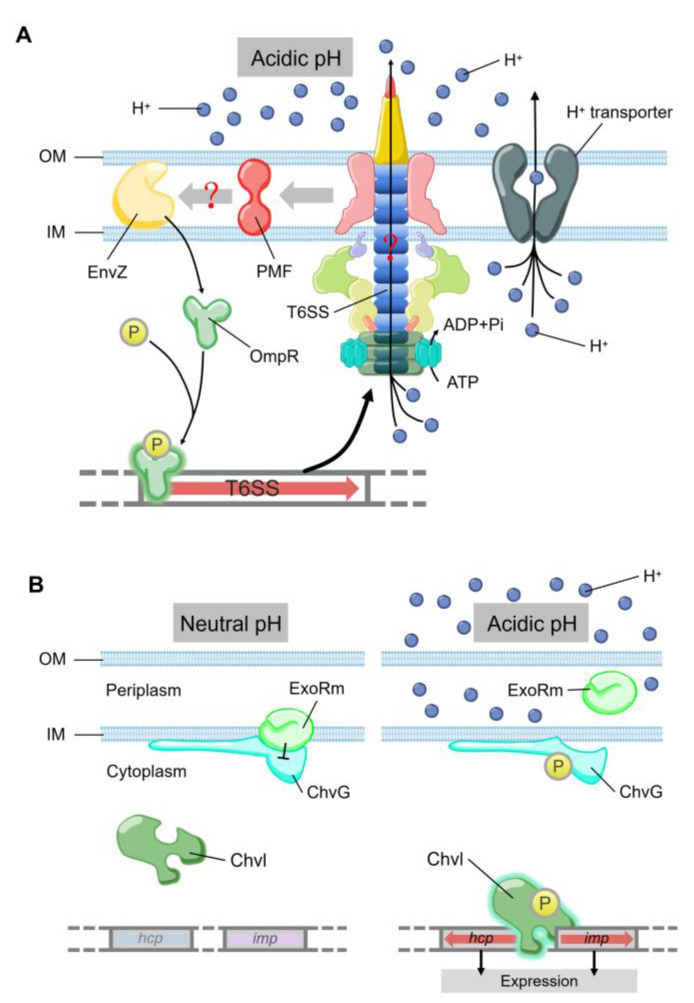
(**A**) The model of T6SS-mediated acid resistance in *Y. pseudotuberculosis.* EnvZ receives the acidic pH signal to promote the phosphorylation of OmpR, which activates the expression of T6SS. T6SS maintains the pH homeostasis by H^+^ extrusion via the ATPase activity of ClpV4. (**B**) Acidic pH can activate T6SS expression by ExoR-ChvG/ChvI. ExoR associates with ChvG and inhibites the activity of ChvG/Chvl two-component system in neutral pH, which reduces the expression and secretion of T6SS. In acidic pH, ExoR degrades from ChvG sensor kinase and then phosphorylates ChvI, which facilitates the expression of *imp* and *hcp*. The color of arrows represents the level of expression. Blue, no expression. Pink, low expression. Red, normal expression.

Low pH is a factor produced by the host’s defense mechanism when it is infected, which can limit the growth of pathogens. T6SS also plays a role in bacterial stress response towards low intracellular pH, an environmental stress encountered frequently by bacteria during phagocytosis. OmpR is a well characterized regulator from the EnvZ/OmpR two-component regulatory system, which regulates the expression of genes in response to changes in the osmolarity and pH [65]. A recent study showed that *Y. pseudotuberculosis* OmpR directly binds to the promoter of T6SS to regulate its expression [66,67] (Figure 3A). The survival rate of T6SS-deficient strains is significantly reduced under acidic conditions [67]. Additionally, OmpR-induced T6SS expression in a low pH environment is essential for the survival of *Y. pseudotuberculosis* [67]. Compared with the wild type, the survival rate of *Y. pseudotuberculosis ompR* mutant in acidic environment was significantly reduced, which was confirmed by measuring colony-forming unit (CFU) after growing for two hours in pH 4.0 buffer [67]. In *Y. pseudotuberculosis*, ClpV4 may uses its ATPase activity to pump hydrogen ions to extracellular matrix to maintain intracellular pH homeostasis. This could be a general strategy for pathogens to resist acidic environments encountered in phagosomes [67]. In *Agrobacterium tumefaciens*, the ExoR-ChvG/ChvI cascade can activate T6SS in acidic conditions [68] (Figure 3B). ExoR associates with ChvG (transmembrane sensor kinase) in neutral pH environment, which inhibits the ChvG/ChvI two-component system signaling by physical interaction, resulting in decreased expression of T6SS and secretion activity [68]. At acidic pH, ExoR no longer inhibits the activity of ChvG, which phosphorylates the response regulator ChvI and thus positively regulates the expression of T6SS [68]. In α-Proteobacteria (including many symbionts and plant or animal pathogens), it may be a common phenomenon that ExoR-ChvG/ChvI cascade system regulates T6SS due to the extensive distribution and highly conservative nature of ExoR and ChvG/ChvI in these bacteria.

**Figure 4:**
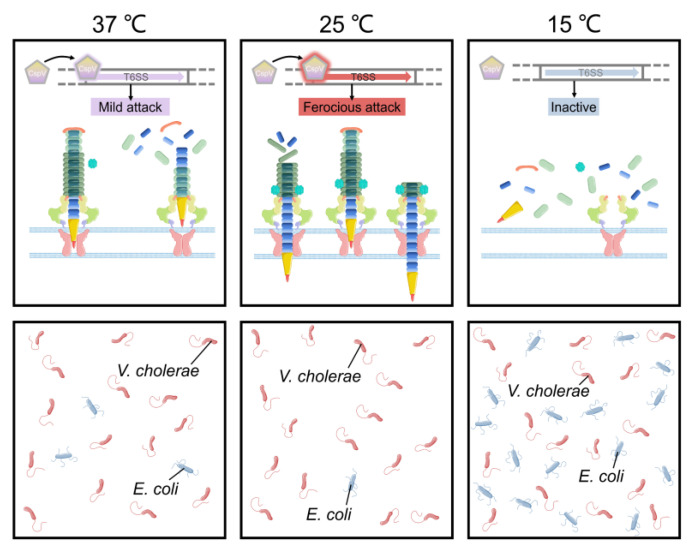
T6SS-mediated temperature adaptation and competition. The expression of T6SS-related genes in *V. cholerae* is regulated by temperature. *V. cholerae* showed significantly different virulence at different temperatures.

*V. cholerae* undergoes temperature fluctuations in its natural aquatic habitat and during the course of infection. Temperature is a key signal to control the outbreak of cholera. Recent studies showed that bacterial pathogens could employ T6SS to facilitate adaptation to temperature changes. For example, temperature is a vital environmental signal to regulate *V. cholerae* physiology and has a huge influence on its survival status and pathogenicity [69]. The *V. cholerae* cold shock protein CspV was shown to regulate T6SS and mediate interspecies killing when temperature is changed [69]. In the aquatic crustacean *Daphnia magna* infection model, CspV-deficient *V. cholerae* strains showed significantly reduced virulence that is mediated by T6SS [69]. The expression of *V. cholerae* T6SS-related genes such as *hcp*, which encodes a hemolysin-coregulated protein, was down-regulated at 15 °C and up-regulated at 25 °C [69,70]. Moreover, using *E. coli* as a prey strain in mixed culture with *V. cholerae* showed that the number of colony-forming unit of *E. coli* decreased by 5-fold at 37 °C, and by 16-fold at 25 °C, without significant change at 15 °C. During this competition process, expression of *CspV* was essential for *V. cholerae* to kill *E. coli* through T6SS [69] (Figure 4). Temperatures of 15 °C and 25 °C simulated the living conditions of *V. cholerae* in the environment and 37 °C simulated the living environment in the human body [69]. Transcriptomic analysis showed that *V. cholerae* T6SS expression varied at different stages of the infection process when it invades the host and enters the environment from the host [69]. *V. cholerae* can respond to changes in temperature, thereby changing the ability to form biofilms and activate T6SS [69]. These processes in turn affect bacterial survival and pathogenicity. Temperature changes may be the key signal for *V. cholerae* to distinguish the host from the environment and to promote the expression of genes essential to survive in different environments. This is important to understanding the mechanism of pathogen survival in the host. In the continuation of the infection cycle, how to re-adapt to the environment after leaving the human body is also an important life process [69,71,72].

## 4. Interspecies and Intraspecies Competition

**Figure 5:**
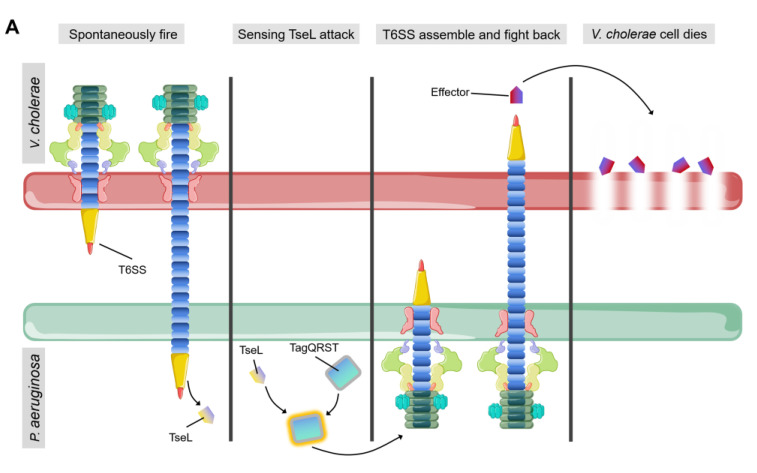

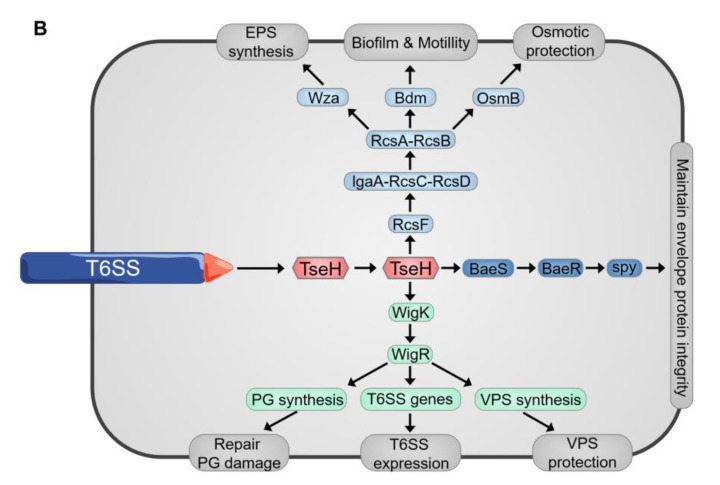
(**A**) Spontaneous firing from *V. cholerae* T6SS and retaliatory attack from *P. aeruginosa*. TagQRST cascade senses T6SS-delivered TseL and induces a counterstrike in *P. aeruginosa*. (**B**) After sensing the effector attack of T6SS, *E. coli* and *V. cholerae* induce envelope stress response through Rcs phosphorelay and BaeSR (blue) or WigKR (green). The induced genes produce protective effects in different ways. PG, peptidoglycan. EPS, exopolysaccharides. VPS, *Vibrio* polysaccharide.

Bacteria can sense attack from T6SS of neighboring cells and activate their own stress response towards them [73]. One of the most well characterized examples is that *P. aeruginosa* rapidly activates its own T6SS to fight against *V. cholerae* after being attacked by TseL (T6SS effector with phospholipase activity) from *V. cholerae* [26,74] (Figure 5A). Kamal and colleague suggested that TseL can activate the expression and assembly of *P. aeruginosa* T6SS to evoke a retaliatory response to *V. cholerae* [74]. Under T6SS attack from neighboring cells, *P. aeruginosa* activates its immunity-independent stress response pathways for self-protection [74]. On the other hand, the regulation cascade of TagQRST-PpkA-PppA-Fha1 can sense attack from *V. cholerae* T6SS [26,74,75,76]. TseL can be sensed by TagQRST which in turn induces *P. aeruginosa* retaliation against *V. cholerae* [26,74]. The stress response system of *P. aeruginosa* coordinates a strong defense against the effectors of *V. cholerae* and contributes to its survival during bacterial competition [74]. This novel type of immunity-independent stress response is similar to the innate immunity in bacteria [74].

Since many T6SS systems attack bacterial cell wall, envelope stress response can often be evoked by bacteria to defend against these attacks [77,78,79] (Figure 5B). Envelope stress response maintains the membrane integrity to resist the damage induced by the effectors [74,79]. The *V. cholerae* TseH, a PAAR-dependent species-specific killing T6SS effector, was shown to damage the cell wall and activate two envelope stress response pathways, Rcs phosphorelay and BaeSR two-component system, of *E. coli* [77,78,79]. Rcs helps *E. coli* to defend against TseH-mediated attacks by activating the expression of osmotic-response genes and increasing the production of exopolysaccharides (EPS) [79]. Moreover, Rcs induces capsule synthesis and the formation of mucoid colonies, where the colanic acid capsule significantly increases *E. coli* resistance to the attack from T6SS [79,80]. *V. cholerae* employs a two-component system WigKR (VxrAB), to respond to cell wall damage and induces the expression of peptidoglycan-repair genes to protect itself from self-killing, as a type of immune-independent Gene defense [79,81,82]. The wild-type *V. cholerae* strain can efficiently kill the *wigKR* mutant by delivering TseH [79]. WigKR positively regulates the formation of biofilms and the synthesis of *Vibrio* polysaccharide (VPS) [83,84]. Interestingly, the presence of *vpsA* has no impact on the sensitivity of *wigR* mutant to its own T6SS, which demonstrates that the protection from WigKR on bacteria is not due to the regulation of VPS but due to superseding VPS [79].

Stress responses are also involved in intra-species competition mediated by T6SS. After two T6SS-expressing *Salmonella* Typhimurium stains, SL1344 (S1) and ATCC14028 (S2), were mixed and cultured, S1 could sense T6SS of S2 and activate RpoS and SoxRS coordinating stress response, including upregulation of expression of *csgD*, *tolC*, and *hilA* genes [73]. Interestingly, when S1 was co-cultured with S2 mutant lacking ClpV (an essential component of T6SS function), the expression of *csgD*, *tolC*, and *hilA* of S1 was no longer up-regulated [73]. *CsgD*, *tolC*, and *hilA* genes are involved in biofilm matrix production, chemical efflux, antibiotic tolerance, and epithelial invasion [73,85,86,87,88,89]. Similarly, *V. cholerae* stains with the same T6SS effector module sets can coexist and be compatible, while strains lacking cognate immunity genes are incompatible and outcompeted [90]. Toxigenic *V. cholerae* strains carry the AAA effector/immunity module [90], which provides the most effective killing of non-kin *V. cholerae* strains [91]. Interestingly, the predator *V. cholerae* strains incorporate the extracellular prey DNA released from the bacteria killed by T6SS. Therefore, Kostiuk and colleagues proposed a model: *V. cholerae* can exchange genetic information including T6SS effector modules during competition in the environment and generate a diverse genotypic pool with members of the same species, similar to genetic card reshuffling [91].

## 5. Involvement of T6SS in the Regulation of Host Immune Signaling Pathways

Inflammasome activation is a crucial defense mechanism of innate immune response, which is used by the host to fight against invading bacteria [92,93]. The host can trigger pyroptosiss by releasing pro-inflammatory cytokines during the activation of inflammasomes [94,95]. Inflammatory cytoplasmic content is released into extracellular environment after pyroptosis of myeloid cells, which further activates innate immune response and accelerates the clearance of bacteria in vivo [93,96]. *Edwardsiella tarda* can significantly inhibit the formation of NLRP3 inflammasome via T6SS [97]. EvpP (T6SS effector of *Edwardsiella tarda*) can inhibit Ca^2+^-dependent c-Jun N-terminal kinase (Jnk, a stress-responsive MAPK signaling pathway) activation, which results in the failure of ASC oligomerization and ultimately inhibits the activation of NLRP3 inflammasome [97]. The inhibition of EvpP-mediated NLRP3 inflammasome notably promoted bacterial colonization in vivo [97]. This is a mechanism adopted by pathogenic bacteria to block host signal transduction and evade from the innate immune response of the host [97].

The highly motile phagocytic cells, neutrophils and macrophages, can be recruited to the sites of tissue infection as the first-line of defence [98]. Morpholino can be used to further analyze the formation of inflammasome in neutrophils recruitment in zebrafish [99]. Neutrophils and macrophages are necessary for zebrafish to combat *E. piscicida* proliferation and infection. After using *caspy*- or *IL-1β*-morpholino knockdown larvae to suppress the development of neutrophils and macrophages in zebrafish larvae and comparing with that of control zebrafish, the survival rate of zebrafish larvae decreased significantly when it was infected by *E. piscicida* [99]. The T6SS effector EvpP inhibits the recruitment of neutrophils to promote *E. piscicida* proliferation and infection [99]. Furthermore, it is EvpP inhibiting the phosphorylation of Jnk-MAPK signal pathway that leads to the inhibition of neutrophils recruitment since the MAPK signal cascade can regulate the activation of gene transcription of a variety of proinflammatory and chemokines [99,100,101]. EvpP regulates the expression of ligand 8 (cxcl8a, also known as IL-8) and matrix metallopeptidase 13 (mmp13) by inhibiting the Jnk-MAPK signal cascade [99]. In zebrafish, cxcl8 is the most effective chemokine which guides neutrophils through the tissue matrix to reach the site of injury or infection [102]. Mmp13 also plays an important role in the recruitment of neutrophils [101]. The Jnk-MAPK signaling pathway plays an important role in *E. piscicida* clearance, neutrophil migration and downstream inflammasome activation in zebrafish [99]. EvpP inhibits the activation of the Jnk-caspy inflammasome pathway in zebrafish larvae, which in turn inhibits the recruitment of neutrophils and highlights the key role of myeloid cells in coping with *E. piscicida* infection in zebrafish [99].

Hemolysin-coregulated protein (Hcp) of *Salmonella enteritidis* is a structural component of the T6SS forming the inner tube which is critical to the secretion of effector proteins and the assembly of T6SS apparatus [103,104]. Subcellular localization of Hcp in the cytoplasm was discovered by expressing plasmid carrying pEGFP-N1-hcp in BHK-21 cells, where Hcp regulates the gene expression along the TNF signaling pathway [105].

## 6. Conclusions

The widely distributed T6SS helps bacteria not only in getting a competitive advantage, but also adapting to various stress conditions. Increasing numbers of T6SS effectors and immunity proteins are unraveled and crystal structures are also solved for many of these proteins. Current studies show that bacteria can take up metal ions through T6SS to resist the damage caused by oxidative stress. Furthermore, T6SS is involved in bacterial adaptation to temperature and pH changes, while T6SS immunity proteins protect self or kin cells from the effector toxicity. Recent studies show that non-self cells can use AID systems to neutralize T6SS toxins. Thus, T6SS functions inclusively as a toxin secretion system for getting interspecies and intraspecies advantages and also a strategy for resisting external pressures and mediate stress responses. The mode of action of many T6SS effectors is still unknown and the discovery of more T6SS effectors is ongoing. Interestingly, there are also evidences showing a high relevance between T6SS bacteria and the progression of host infection. We hypothesize that T6SS mediated stress response mechanisms are widely adopted by bacterial pathogens during interspecies competition as well as environmental adaptation. Further clarification of T6SS mediated stress response mechanisms may help us to understand microbial ecology, manipulation of the compositions of microbial communities, and develop novel antimicrobial strategies. T6SS could have great potential application in synthetic biology. The establishment of synthetic T6SS in a probiotic or controllable tool to modulate host immunity and control intestinal inflammation, in order to regulate host immunity or reshape microbial structure and function, will be an interesting topic. More and more evidence points to T6SS as a comprehensive survival strategy adopted by Gram-negative bacteria. Another topic worthy of discussion is the potential role of T6SS in signal transduction among microbial communities. The current research in the field still leaves great space for the discovery of T6SS effectors. Furthermore, the exploration of their biological functions is another exciting and significant topic of research.

## Data Availability

Not applicable.
